# Mapping the methodological framework of diabetic retinopathy interventional trials: a scoping review on trial design, execution, and reporting practices

**DOI:** 10.3389/fendo.2026.1961622

**Published:** 2026-10-09

**Authors:** Zixuan Wang, Xuwei Liu, Ming Yang, Guanchi Yan, Xuechun Fan, Jing Yu, Jinyue Zhao, Yingjing Shi, Xi Chen

**Affiliations:** 1 College of Traditional Chinese Medicine, Changchun University of Chinese Medicine, Changchun, China; 2 School of Environment and Energy, Beijing University of Civil Engineering and Architecture, Beijing, China; 3 The Affiliated Hospital to Changchun University of Chinese Medicine, Changchun University of Chinese Medicine, Changchun, China; 4 Changchun Disabled Persons Rehabilitation Center, Changchun, China

**Keywords:** clinical trial, diabetic macular edema, diabetic retinopathy, intervention, scoping review

## Abstract

**Background:**

Diabetic Retinopathy (DR) is a prevalent and sight-threatening ocular complication of diabetes, and one of the leading causes of vision loss and blindness among the working-age population worldwide. Driven by a growing understanding of the pathophysiological mechanisms underlying DR, treatment strategies have evolved from a sole focus on glycemic control to comprehensive, multi-targeted, and multimodal therapies. This study systematically summarizes the characteristics of current clinical trials focused on DR treatment and evaluates the employed research methodologies. The aim of this review is to explore methodological trends in specific domains, with the hope that such insights may inform future efforts to optimize trial design and improve trial quality.

**Methods:**

This study followed the PRISMA-ScR reporting guidelines. Clinical trials registered on ClinicalTrials.gov and the Chinese Clinical Trial Registry (ChiCTR) were retrieved. In addition, clinical trials indexed in PubMed from July 23, 2016 to July 23, 2026 were retrieved. Data from the included studies were extracted and presented in tabular form.

**Results:**

Clinical research targeting DR has expanded rapidly, reflecting a shift toward diverse interventions like anti-Vascular endothelial growth factor(anti-VEGF) drugs, laser treatments, and combined therapies. However, several methodological limitations were noted in the included studies, including the failure to specify diabetes type in over half of the cases, a predominance of small-sample trials, inconsistent reporting of intervention duration, and limited implementation of blinding due to practical constraints in ophthalmic interventions.

**Conclusion:**

This review represents the first comprehensive assessment of current DR clinical research, exposing key design weaknesses in existing trials. Future meta-analyses and systematic evaluations, informed by the methodological gaps identified in this review, are urgently needed to generate robust evidence that can guide optimal treatment strategies.

## 1 Introduction

Diabetic Retinopathy (DR), a predominant microvascular complication of diabetes, stands as a leading cause of preventable blindness among the global working-age population (1). Epidemiological data indicate that the global prevalence of DR among individuals with diabetes ranges from 21.95% (1) to 31.60%, with Vision-threatening Diabetic Retinopathy (VTDR) accounting for 5.06% of these cases. A 2021 analysis of United States data revealed a DR prevalence of 26.43% (2). Notably, significant geographical heterogeneity exists; the highest prevalence rates are observed in Africa and the Middle East. These disparities correlate strongly with regional variances in diabetes management standards and healthcare accessibility (3).

Regarding incidence, the cumulative risk of developing DR within five years of a type 2 diabetes diagnosis is approximately 10–15%, a rate that escalates exponentially with disease duration. In type 1 diabetes, a Saudi Arabian study reported an incidence density of 92.66 per 1,000 person-years (4). Moreover, a 20-year retrospective cohort study in the United States documented a steady annual increase in DR prevalence post-2007, reaching 26.43% by 2021 (2). Collectively, these statistics underscore a substantial and unevenly distributed global burden. Pathophysiologically, DR typically evolves from asymptomatic early Non-proliferative Diabetic Retinopathy (NPDR) to advanced Proliferative Diabetic Retinopathy (PDR), characterized by pathological neovascularization and sight-threatening sequelae such as vitreous hemorrhage and tractional retinal detachment. Concurrently, Diabetic Macular Edema (DME), characterized by increased vascular permeability and retinal thickening, may manifest at any stage of the disease, presenting a distinct threat to visual acuity (5).

Historically, DR management prioritized systemic optimization of glycemic, blood pressure, and lipid profiles (6, 7). While these interventions are proven to mitigate risk and decelerate progression, they offer incomplete protection, implicating auxiliary pathogenic mechanisms such as genetic susceptibility and local ocular factors (8, 9). Ophthalmic intervention has traditionally relied on laser photocoagulation—specifically Panretinal Photocoagulation (PRP) for PDR and focal/grid laser for DME—to ablate ischemic retina and stabilize edema. Although laser therapy remains effective in preventing severe vision loss, it is not without potential adverse sequelae (10, 11).

By inhibiting neovascularization and resolving macular edema, anti-VEGF therapy significantly improves visual prognosis (10). Recent randomized clinical trials demonstrate that early intervention with intravitreal anti-VEGF injections in moderate-to-severe NPDR reduces the incidence of vision-threatening complications and improves DR severity scores. This evidence marks a paradigm shift from the traditional “watch-and-wait” approach to proactive pharmacological management (12, 13). However, consensus regarding the optimal timing, dosing frequency, and treatment duration remains elusive. Furthermore, the necessity for repeated intravitreal injections presents significant challenges regarding patient adherence and economic burden (8).

Emerging pathophysiological insights highlight the critical roles of inflammation and neurodegeneration in DR. Elevated levels of systemic and intraocular inflammatory mediators—including interleukins (IL-6, IL-8), interferon-gamma, and tumor necrosis factor-alpha—correlate closely with disease severity and DME presence, underscoring the inflammatory component of DR progression (14, 15). Concurrently, diminished levels of neurotrophic factors, such as brain-derived neurotrophic factor (BDNF), have been identified in NPDR and DME patients. This suggests that neurodegenerative processes not only contribute to retinal dysfunction but may also represent viable therapeutic targets (16).

The scientific validity and clinical applicability of research findings are inextricably linked to the rigor of clinical trial design (17). Transparent trial registration is pivotal for mitigating research waste, preventing duplication, and optimizing resource allocation, thereby elevating the overall quality of clinical evidence (18).

This review synthesizes data from ClinicalTrials.gov, the Chinese Clinical Trial Registry (ChiCTR), and PubMed. While existing literature reviews address DR treatment and diagnosis, there is a paucity of systematic analyses focusing specifically on the methodology of interventional clinical trials (19, 20). To the best of our knowledge, this study attempts to clarify the main questions driving this review by focusing on three specific methodological domains: design rigor, reporting completeness, and outcome selection. The evaluation of design rigor and protocol completeness was guided by the SPIRIT 2025 statement (21). It is hoped that the findings from these databases may provide insights into potential areas for optimizing experimental design, which could be beneficial for future DR clinical research.

## 2 Methods

### 2.1 Search strategy

This study adhered to the PRISMA-ScR reporting guidelines. The study was conducted to identify relevant clinical trials published between July 23, 2016, and July 23, 2026. This ten-year window was selected to capture the contemporary landscape of diabetic retinopathy (DR) research, specifically reflecting the period following the widespread establishment of anti-VEGF therapy as the standard of care and the maturation of large-scale multicenter trial networks (22).

For the PubMed database, the search strategy was constructed using a combination of Medical Subject Headings (MeSH) and free-text terms. As detailed in **Supplementary Table 1**, synonyms for “Diabetic Retinopathy” (including MeSH Major Topic and title/abstract keywords) were combined using the Boolean operator “OR” to maximize sensitivity. This search set was then combined with study type filters for “Clinical Trial” and “Randomized Controlled Trial” using the Boolean operator “AND” to ensure specificity. Similar logic involving controlled vocabulary and keyword combinations was applied to other databases where applicable.

### 2.2 Inclusion and exclusion criteria

The inclusion criteria were as follows: 1) Study subjects: Patients with diabetic retinopathy (DR); 2) Interventions: Evaluation of any intervention primarily aimed at treatment, including anti-VEGF, laser, surgery, hormones, and ethnomedicine treatments; 3) Study types: Interventional clinical trials, including randomized controlled trials (RCTs), non-randomized controlled trials (non-RCTs), and single-arm trials; 4) Time frame: The search strategy encompassed full-time data from the ClinicalTrials.gov and ChiCTR registries, whereas PubMed searches were restricted to a ten-year window from July 23, 2016, to July 23, 2026.

Exclusion criteria are as follows: 1) Studies unrelated to diabetic retinopathy; 2) Non-clinical trials (basic research, reviews); 3) Studies for which the full text was inaccessible or data could not be extracted.

### 2.3 Data extraction

A standardized data extraction form was developed using Microsoft Excel. Prior to formal data collection, two reviewers (Zixuan Wang and Xuwei Liu) conducted a pilot extraction on a random subset of the included studies. Inter-reviewer reliability was assessed using Cohen’s kappa statistic for categorical variables and intraclass correlation coefficient (ICC) for continuous variables, with percent agreement also reported. A kappa value of ≥ 0.80 and an ICC of ≥ 0.75 were considered indicative of substantial to excellent agreement. Following the pilot phase, the extraction form and protocols were refined based on these assessments. The finalized form captured key variables, including the number of registrations or publications, country, intervention type, participant characteristics, primary and secondary outcomes, and study design; variables not captured by the predefined categories were recorded under “Other.” Formal data extraction was then performed independently by the two reviewers. The extracted datasets were cross-verified to identify discrepancies. Any discrepancies were resolved by re-examining the original sources to reach consensus. In cases where agreement could not be reached, a third senior researcher (Xi Chen) was consulted to make the final adjudication.

### 2.4 Statistical analysis

Data analysis and processing were conducted in Excel. As this study is a comprehensive review of clinical research on diabetic retinopathy, descriptive statistical methods were used to summarize the study characteristics extracted from the included literature using frequencies and percentages.

## 3 Results

### 3.1 Study selection and inclusion criteria

A total of 707 records were included in this study, comprising 450 registrations from ClinicalTrials.gov, 71 registrations from the Chinese Clinical Trial Registry (ChiCTR), and 186 publications indexed in PubMed. A total of 1,540 records were initially retrieved from these three databases. To address potential overlaps between publications and registry entries, we implemented a systematic deduplication process. We first conducted a preliminary screen by extracting author names and NCT or ChiCTR registration numbers mentioned in the abstracts of included studies to facilitate duplicate identification. Specifically, we further deduplicated the records as follows: 1) Records with identical study identifiers (NCT numbers or ChiCTR numbers) extracted from abstracts were merged into single entries; 2) Records with matching author names across different databases were manually reviewed to identify duplicates, which were then retained as single entries; 3) Within each database, records were screened for exact and near-duplicate matches. When duplicates were identified across databases, registry records were prioritized over corresponding publications. A total of 113 duplicate records were removed through this multi-step process. Next, the remaining records were screened by title and abstract, resulting in the exclusion of 636 irrelevant studies. Finally, full-text reviews were conducted on the remaining records, leading to the exclusion of 82 studies that did not meet the inclusion criteria. Furthermore, through detailed study comparison and matching, 2 studies were identified as highly overlapping and were subsequently excluded. Thus, this study ultimately included 707 records. The literature screening process was conducted in accordance with the PRISMA guidelines, as illustrated in **Figure 1** (23, 24). Detailed search results are provided in **Supplementary Table 2**.

**Figure 1:**
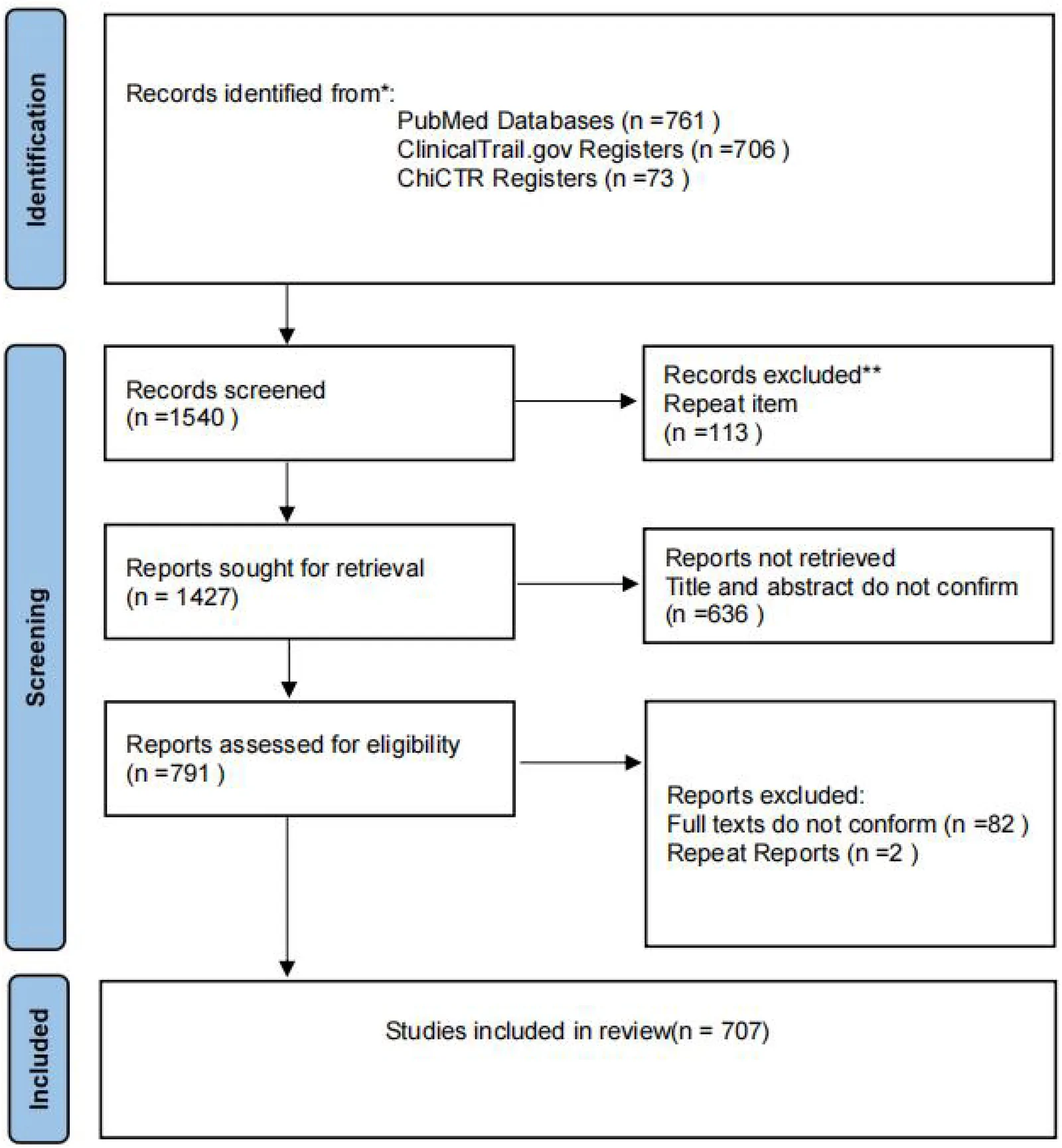
PRISMA search flowchart.

### 3.2 Number of registrations and publications, country

The temporal trend of clinical research shows that studies on DR commenced an upward trend in 2005, maintained steady growth, and then experienced a substantial surge from 2018, ultimately attaining its peak. Research activity sustained a high level from 2019 to 2025 and is projected to remain robust through 2026. The annual evolutionary trends of DR are illustrated in **Figure 2**.

**Figure 2:**
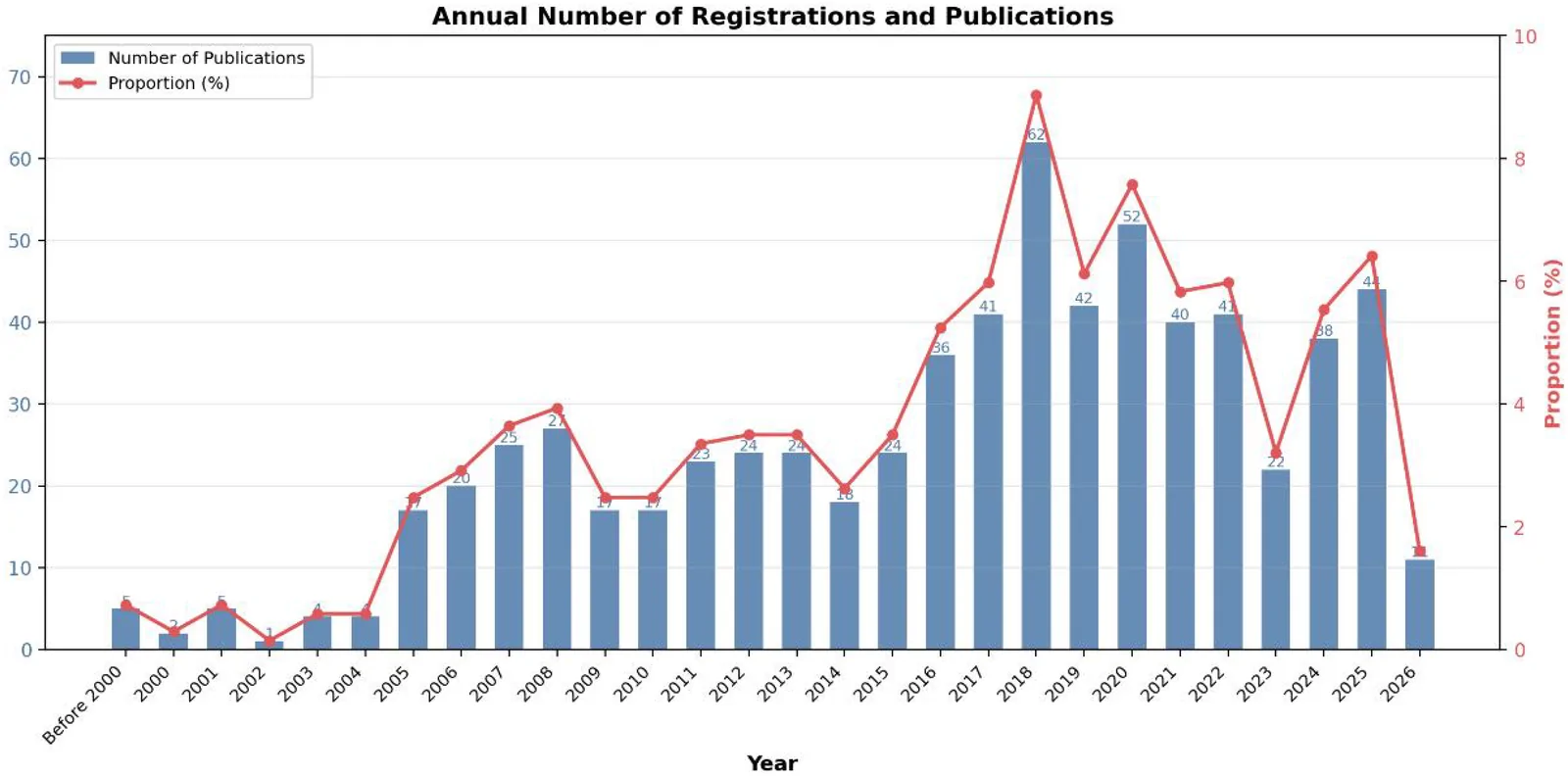
Annual number of registrations and publications: abscissa represents the year, left ordinate represents the proportion, and right ordinate represents the number.

Geographically, the United States leads in the absolute number of trials (177 studies, 25.04%), with a significant portion of high-quality data originating from the DRCR.net, which has been pivotal in shaping clinical guidelines. China ranks second (143 studies, 20.23%), with a notable focus on traditional Chinese medicine and plant extracts. European nations also made substantial contributions, including the UK (40 studies, 5.66%), Germany (38 studies, 5.37%), Spain (27 studies, 3.82%), France (26 studies, 3.68%), Italy (25 studies, 3.54%), and Hungary (20 studies, 2.83%). Other key contributors include Japan (32 studies, 4.53%), Brazil (29 studies, 4.10%), Mexico (29 studies, 4.10%), Canada (25 studies, 3.54%), Australia (23 studies, 3.25%), and Iran (20 studies, 2.83%). The global distribution of these trials is illustrated in **Figure 3**.

**Figure 3:**
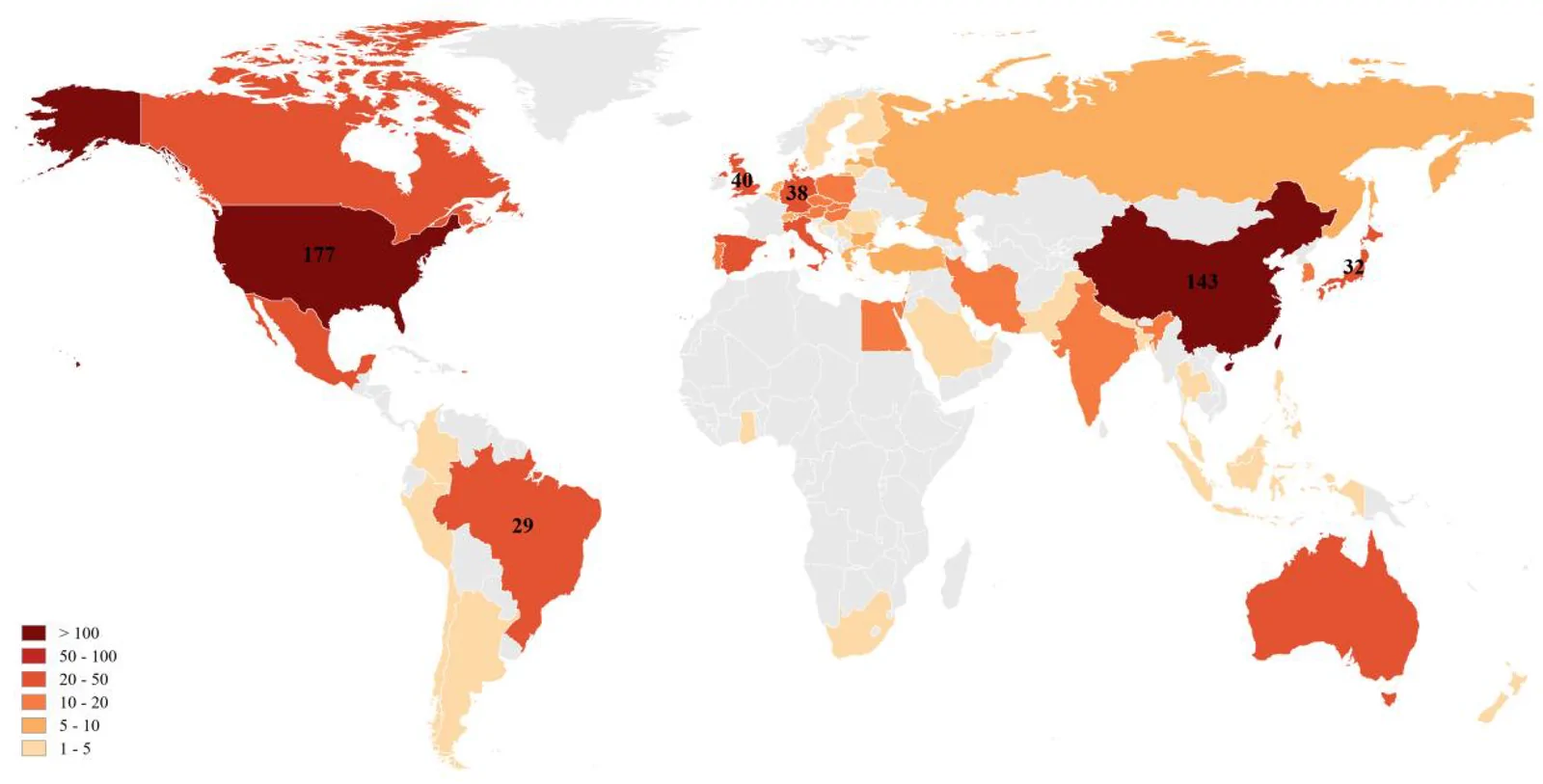
Published or registered country.

### 3.3 Classification of intervention measures characteristics

Anti-VEGF therapy was the most prevalent intervention, featured in 175 studies (24.75%)—nearly one-quarter of the total. Perioperative interventions (104 studies, 14.71%) and combination therapies (92 studies, 13.01%) followed in frequency. Together, these three categories accounted for over half (52.48%) of all studies.

The second tier of interventions included laser therapy (48 studies, 6.79%), comparative effectiveness studies (47 studies, 6.65%), and corticosteroid-based pharmacotherapy (44 studies, 6.22%). The remaining categories were more fragmented, each representing less than 5% of studies. These included traditional Chinese medicine (4.38%), lifestyle interventions (4.10%), ophthalmic surgery (3.39%), systemic metabolic control (3.39%), eye drops (2.40%), nutritional supplements (2.40%), anti-inflammatory agents (2.26%), and targeted therapies (1.70%). Detailed characteristics of interventions are shown in **Figure 4**. The 39 intervention categories listed in **Supplementary Table 3** are mutually exclusive. Each study was assigned to only one category based on its primary intervention focus. When a study involved multiple intervention modalities, the predominant or defining intervention was used to determine the single applicable category.

**Figure 4:**
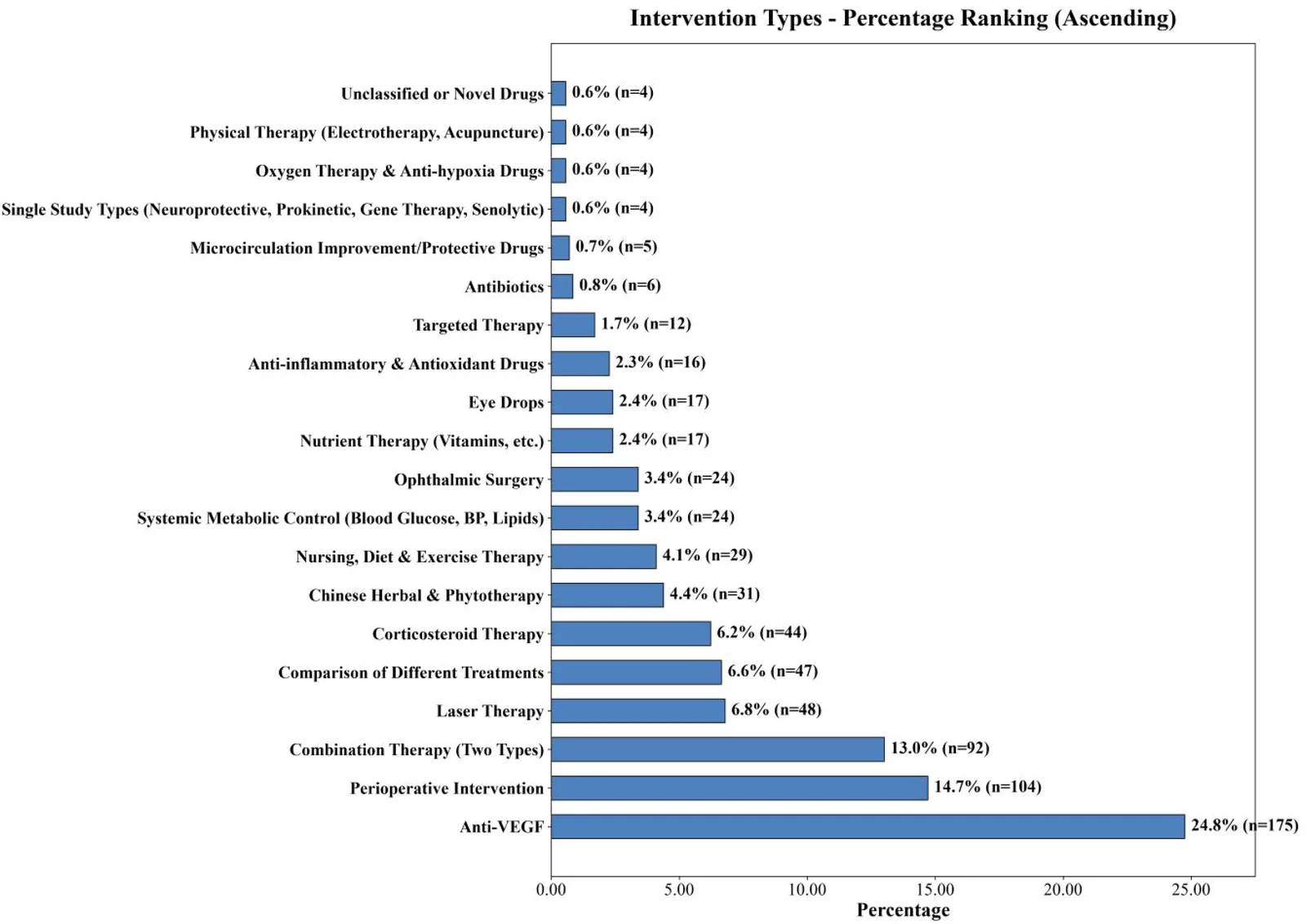
Classification of interventions.

Among the 92 studies on combination therapy, regimens were heavily skewed toward anti-VEGF agents. The combination of anti-VEGF therapy and laser photocoagulation was the most common (42 studies, 45.65%). The combination of anti-VEGF therapy and corticosteroids ranked second (17 studies, 18.48%). Together, these two regimens constituted the primary tier of combination approaches, accounting for 64.13% of such studies. Detailed characteristics of combination therapy are shown in **Figure 5**.

**Figure 5:**
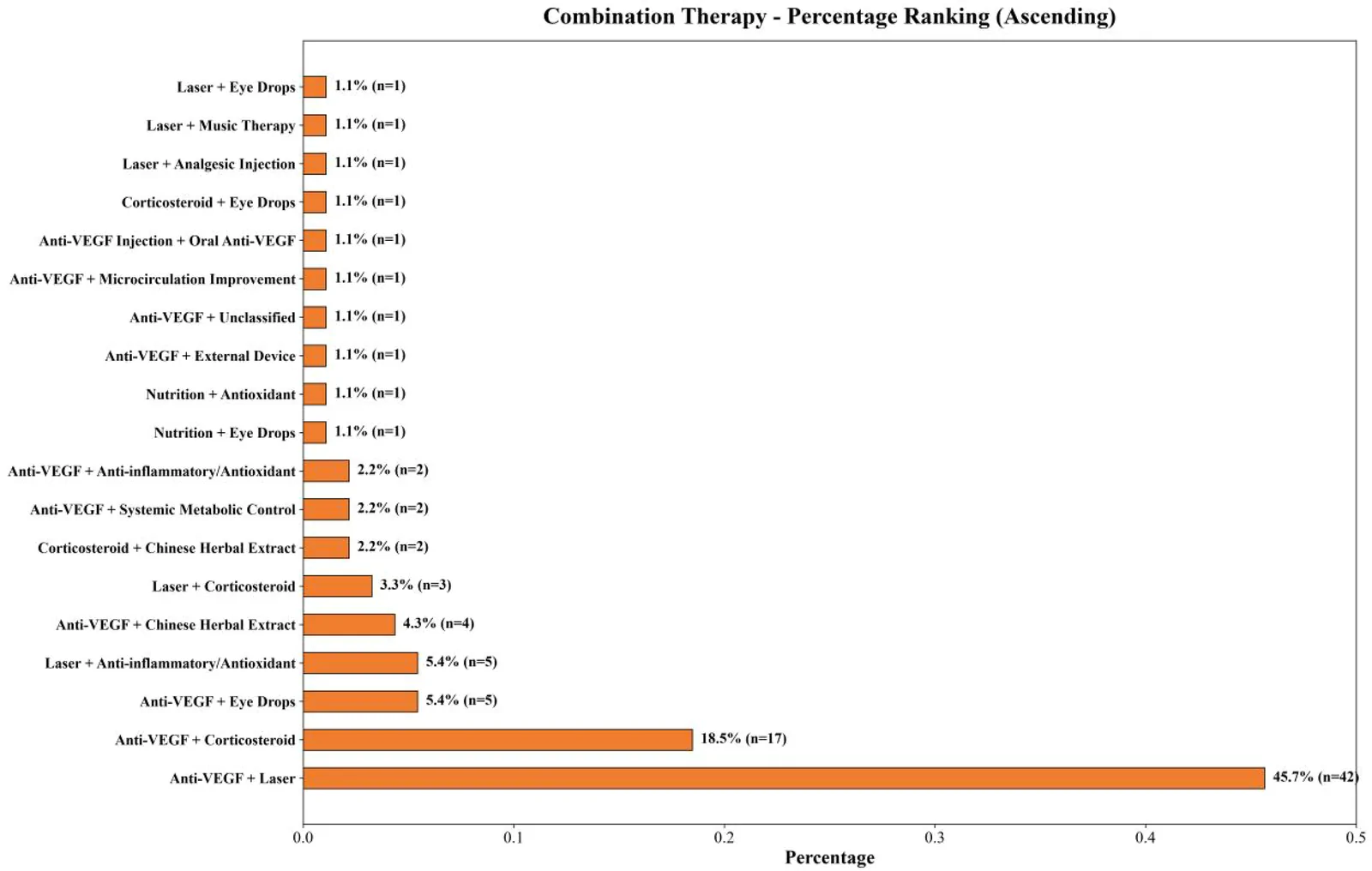
Classification of combination therapy.

### 3.4 Study population and basic characteristics of diabetic retinopathy

Among the 707 studies, over half (364 studies, 51.49%) did not specify the type of diabetes, referring to it only in general terms. Of those that did, 245 studies (34.65%) distinguished between type 1 and type 2 diabetes; separately, 88 studies (12.45%) focused exclusively on type 2 diabetes, while only 10 (1.41%) were limited to type 1 diabetes.

Regarding DR classification, PDR was the most frequently studied stage (197 studies, 27.86%), followed by NPDR (136 studies, 19.24%). Macular edema was a key feature in 51.63% of studies (365 studies), with Center-involved Diabetic Macular Edema (CI-DME) being the most specified subtype (208 studies, 29.42%).

Most studies (89.96%) established clear ophthalmic examination criteria. However, fewer specified glycemic control criteria (25.32%) or ancillary examinations (27.58%). In terms of visual impairment, most enrolled patients had early-to-moderate disease, with mild (BCVA <20/25; 10.61%) and moderate (BCVA <20/40; 10.33%) decreases being the most common. Central retinal thickness (CRT) was assessed in 21.78% of all included studies, with >300μm being the most common inclusion threshold (10.89%). **Table 1** details the characteristics of the study populations.

**Table 1:** Characteristics of intervention population.

Characteristics	Category	Included literature	Record percentage(%)
Types of Diabetes	Only diabetes	364	51.49%
	Both Type 1 and Type 2 diabetes	245	34.65%
	Type 2 diabetes	88	12.45%
	Type 1 diabetes	10	1.41%
Retinopathy Type	NPDR	136	19.24%
	PDR	197	27.86%
	Only DR	79	11.17%
	Only Macular Edema	320	45.26%
Macular Edema Type	All Types Of Macular Edema	365	51.63%
	CI-DME	208	29.42%
	NCI-DME	3	0.42%
	Only DME	154	21.78%
Specific Inspection Requirements Classification	Ophthalmic examination	636	89.96%
	Glycemic and lipid metabolism indicators	179	25.32%
	Other laboratory indicators	195	27.58%
Assessment Of Vision	Total Number Of Vision Tests	295	41.73%
	Total Decline In Vision	152	21.50%
	Mild decrease in visual acuity (best corrected visual acuity on Snellen test <20/25)	75	10.61%
	Moderate visual impairment (best corrected visual acuity on Snellen test <20/40)	73	10.33%
	Significantly decreased visual acuity (best corrected visual acuity on Snellen test <20/80)	1	0.14%
	Legal blindness (best corrected visual acuity per Snellen test <20/200)	3	0.42%
Assessment of central retinal thickness in patients with macular edema	Total number of evaluations for diabetic macular edema	154	21.78%
	>300μm	77	10.89%
	>250μm	25	3.54%
	Dynamic parameters	16	2.26%
	>320μm	13	1.84%
	>275μm	7	0.99%
	>350μm	5	0.71%
	>400μm	4	0.57%
	>325μm	3	0.42%
	>200μm	1	0.14%
	>225μm	1	0.14%
	>260μm	1	0.14%
	>310μm	1	0.14%
Ophthalmic Features	Cataract	19	2.69%
	Hard Exudates	6	0.85%
	Tractional Retinal Detachment	25	3.54%
	Vitreous Hemorrhage	27	3.82%

DR, Diabetic Retinopathy; NPDR, Non-Proliferative Diabetic Retinopathy; PDR, Proliferative Diabetic Retinopathy; CI-DME, Center-involved Diabetic Macular Edema; DME, Diabetic Macular Edema; NCI-DME, Non-Center-involved Diabetic Macular Edema. Dynamic parameters: Zeiss Cirrus – 275 μm for females, 290 μm for males; Heidelberg Spectralis – 290 μm for females, 305 μm for males. Data are presented as number (percentage). Percentages are calculated based on the total number of included studies (n=707) unless otherwise specified. Retinopathy Type, Macular Edema Type, Specific Inspection Requirements Classification, and Ophthalmic Features are not mutually exclusive; all other categories are mutually exclusive. The classification of diabetic retinopathy severity (NPDR, PDR) across included trials was primarily based on the Early Treatment Diabetic Retinopathy Study (ETDRS) classification system.

### 3.5 Basic characteristics of clinical trials

The included studies were predominantly small, with 61.81% enrolling fewer than 100 participants. Studies with 1–50 participants were the most common (39.18%), followed by those with 51–100 participants (22.63%). Large-scale trials with over 300 participants accounted for only 13.15%.

Adults constituted the primary study population (63.79%), and nearly all studies (99.86%) included both sexes. Reporting on intervention duration was inconsistent, with 37.48% of studies failing to specify it. Among those that did, a single intervention session was most common (23.62%), consistent with the paradigm for procedures like intravitreal injections. Follow-up duration was reported more consistently, with medium-term follow-up (181–365 days) being the most frequent (26.59%). Overall, most studies (47.95%) had follow-up periods between three months and one year, aligning with the conventional window for efficacy assessment. The basic characteristics of the Clinical trials included in the study are detailed in **Table 2**.

**Table 2:** Basic characteristics of clinical trials.

Characteristics	Category	Included literature	Record percentage(%)
Sample size	Cases 1 to 50	277	39.18%
	Cases 51 to 100	160	22.63%
	Cases 101 to 200	110	15.56%
	Cases 201 to 300	40	5.66%
	Cases over 300	93	13.15%
	Case size not specified/Case size is 0	27	3.82%
Age	Adults (lower age limit: 17–21 years, varying by study)	451	63.79%
	Over 25 years	7	0.99%
	Over 30 years	16	2.26%
	Over 35 years	6	0.85%
	Over 40 years	24	3.39%
	Over 45 years	7	0.99%
	Over 50 years	7	0.99%
	Over 55 years and older(Older Adult)	6	0.85%
	Not labeled/Only labeled for all age groups	183	25.88%
Sex	No gender restrictions	706	99.86%
	Only men	1	0.14%
Intervention Time	No intervention time reported	265	37.48%
	Once	167	23.62%
	1 to 15 days	21	2.97%
	16 to 30 days	18	2.55%
	31 to 90 days	89	12.59%
	91 to 180 days	48	6.79%
	181 to 365 days	63	8.91%
	Over 365 days	36	5.09%
Follow-up Time	No follow-up time reported	82	11.60%
	1 to 30 days	52	7.36%
	31 to 90 days	115	16.27%
	91 to 180 days	151	21.36%
	181 to 365 days	188	26.59%
	Over 365 days	119	16.83%

Data are presented as number (percentage). Percentages are calculated based on the total number of included studies (n=707) unless otherwise specified. All categories are mutually exclusive.

### 3.6 Outcome indicator characteristics

Retinal functional outcomes (e.g., visual acuity) were the most common primary endpoint (324 studies, 45.83%), followed by retinal anatomical parameters (e.g., CRT, CMT) (181 studies, 25.60%). Together, these “function plus structure” endpoints accounted for over 70% of primary outcomes. Safety outcomes (17.11%) and DR progression (13.86%) were the next most frequent. The distribution of secondary endpoints was similar but more diverse. Retinal functional outcomes remained the most prevalent (39.18%), while the proportion of studies reporting anatomical parameters increased from 25.60% to 33.95%. **Table 3** summarizes the distribution of primary, secondary, and safety outcome measures across the included studies.

**Table 3:** Outcome indicator characteristics.

Characteristics	Category	Included literature	Record percentage (%)
Main efficacy indicators	Retinal function indicators	324	45.83%
	Retinal anatomical features	181	25.60%
	Safety indicators	121	17.11%
	Outcome indicators of diabetic retinopathy progression	98	13.86%
	Retinal vascular and circulatory characteristics	70	9.90%
	Laboratory indicators	62	8.77%
	Treatment-related indicators such as number of treatments, treatment duration, and treatment frequency	56	7.92%
	Questionnaire	37	5.23%
	Ophthalmic characteristics	36	5.09%
	Difficult to classify	20	2.83%
	Physical examination	10	1.41%
	Cost-effectiveness	4	0.57%
Secondary efficacy endpoints	Retinal functional indicators	277	39.18%
	Retinal anatomical characteristics	240	33.95%
	Safety indicators	172	24.33%
	Outcome indicators of diabetic retinopathy progression	114	16.12%
	Treatment-related indicators such as number of treatments, treatment duration, and treatment frequency	109	15.42%
	Retinal vascular and circulatory characteristics	88	12.45%
	Laboratory indicators	76	10.75%
	Questionnaire	69	9.76%
	Ophthalmic characteristics	57	8.06%
	Difficult to classify	47	6.65%
	Physical examination	24	3.39%
	Cost-effectiveness	12	1.70%
Safety Indicator	Adverse reactions/Adverse events	240	33.95%
	Physical examination	57	8.06%
	Laboratory Parameters	7	0.99%
	Mark only “Safety Indicator”	19	2.69%

Data are presented as number (percentage). Percentages are calculated based on the total number of included studies (n=707) unless otherwise specified. Categories are not mutually exclusive. Functional outcomes were defined as measures of visual function, primarily best-corrected visual acuity (BCVA). Anatomical outcomes were defined as structural measures of retinal morphology assessed by optical coherence tomography (OCT), including central retinal thickness (CRT), central macular thickness (CMT), intraretinal fluid (IRF), and subretinal fluid (SRF).

### 3.7 Characteristics of clinical trial design

RCTs were the predominant design (70.58%). Parallel-group designs were most common (75.95%), reflecting the focus on comparing therapeutic efficacy between groups. Blinding implementation varied significantly; open-label or partially blinded studies were the most frequent (30.55%), largely due to the practical challenges of blinding ophthalmic procedures. Single-center studies constituted the majority (51.34%), indicating that research remains largely institution-based. Active treatment controls were the most common comparator (59.41%), followed by placebo controls (23.06%), which were typically used in exploratory or add-on studies. Two-arm designs were the standard configuration (62.23%). The methodological features of the included studies are summarized in **Table 4**.

**Table 4:** Characteristics of clinical trial design.

Characteristics	Category	Included literature	Record percentage (%)
Allocation	Randomized	499	70.58%
	Non-randomized	56	7.92%
	Queue/Case-Control/Case-Only	46	6.51%
	Unknown	106	14.99%
Intervention Model	Parallel	537	75.95%
	Single Group Assignment	138	19.52%
	Cross-over Design	15	2.12%
	Sequential Assignment	10	1.41%
	Factorial Design	3	0.42%
	Unknown	4	0.57%
Blind method: Implementing blindness	Open-label (no blinding)/partially open	216	30.55%
	Single blind	97	13.72%
	Double blind	100	14.14%
	Triple Blind	62	8.77%
	Quadruple blind	49	6.93%
	Unknown/Unmarked	183	25.88%
Research Center	Single-center study	363	51.34%
	Multicenter (2–15 centers)	102	14.43%
	Multicenter (16–30 centers)	30	4.24%
	Multicenter (≥31 centers)	60	8.49%
	Multicenter (unspecified number of centers)	40	5.66%
	Unknown	112	15.84%
Treatment regimen for the control group	Placebo Control	163	23.06%
	Conventional Treatment Control	420	59.41%
Number of experimental groups	Single-arm design	135	19.09%
	Double-arm design	440	62.23%
	Three-arm design	93	13.15%
	Four-arm design	21	2.97%
	Design with more than five arms	14	1.98%
	Unknown	4	0.57%

Data are presented as number (percentage). Percentages are calculated based on the total number of included studies (n=707) unless otherwise specified. All categories are mutually exclusive unless otherwise noted. For the “Treatment regimen for the control group” category, categories are not mutually exclusive; studies may employ both placebo and conventional treatment controls simultaneously.

## 4 Discussion

This study provides a comprehensive overview of the current landscape of clinical trials investigating diabetic retinopathy, thereby providing researchers with a structured reference to better inform future trial design. Prior research in this domain has predominantly centered on screening strategies and outcome prediction (25, 26). To date, however, no systematic review has specifically examined the methodological and design characteristics of interventional clinical trials for diabetic retinopathy. To our knowledge, the present study therefore constitutes the first comprehensive assessment of diabetic retinopathy clinical trial features. Clinical research on diabetic retinopathy began to show an upward trend around 2005, coinciding with the emergence of the anti-VEGF therapeutic era. Following FDA approval of bevacizumab for oncological indications in 2004 and ranibizumab for neovascular age-related macular degeneration in 2006, the ophthalmology community rapidly initiated exploratory intravitreal injection studies. This paradigm shift fundamentally transformed DR/DME treatment, where laser photocoagulation had previously been the sole standard of care (27).This therapeutic shift stimulated substantial clinical investigations into the efficacy, safety, and dosing regimens of anti-VEGF agents. Concurrently, the progressive adoption of optical coherence tomography (OCT) provided a standardized tool for objective quantitative assessment of retinal thickness, lowering the imaging threshold for clinical trials and further contributing to research growth (28).

Between 2005 and 2017, DR clinical research maintained steady growth driven by landmark multicenter trials from the DRCR.net (e.g., Protocol I in 2012 establishing ranibizumab plus laser as standard DME therapy, and Protocol T in 2015 comparing ranibizumab, aflibercept, and bevacizumab) (29, 30). The 2012 FDA approval of aflibercept for DME further expanded the therapeutic armamentarium. Meanwhile, the global diabetic population grew from approximately 151 million in 2000 to 463 million in 2019 (International Diabetes Federation), intensifying the DR/DME burden and expanding the eligible participant pool for clinical research (31).

A temporal analysis of the included studies revealed distinct patterns and notable publication lags between clinical trial registrations and subsequent publications in PubMed. For anti-VEGF therapies, the peak of trial registrations occurred in 2016 (12 studies), whereas the highest number of published studies was recorded two years later in 2018 (12 studies). Similarly, regarding perioperative interventions, the maximum number of registrations was observed in 2007 (8 studies), while the peak in publications did not occur until 2020 (6 studies). For the two types of combination therapies, the publication trend remained relatively stable, with peaks observed in 2017, 2019, 2023, and 2025 (4 studies each), whereas the highest volume of registrations was noted in 2019 (6 studies). These discrepancies in peak years highlight a significant time lag between the initiation of clinical trials and their formal publication, underscoring the importance of registry data in capturing the real-time landscape of DR research.

Research activity has remained at a high level from 2019 through 2025 and is projected to sustain this trajectory into 2026. This reflects multiple converging factors: post-marketing Phase IV studies and indication-expansion trials for newly approved agents (e.g., faricimab, brolucizumab, high-dose aflibercept); next-generation therapeutic strategies—including gene therapy, complement pathway inhibitors, sustained-release formulations, VEGF/FGF dual-target fusion proteins, and neurovascular unit protection strategies—transitioning from preclinical to clinical stages; and the rapid advancement of AI-assisted DR screening, diagnostic technologies, and telemedicine models (32, 33).

Anti-VEGF combined with retinal laser photocoagulation accounted for 45.65% of the main combination regimens. Anti-VEGF drugs (e.g., ranibizumab, aflibercept, bevacizumab) rapidly reduce macular edema and inhibit neovascular activity by neutralizing VEGF, with a fast onset but limited duration of action. Laser photocoagulation, by contrast, destroys high oxygen-consuming photoreceptor cells to reduce retinal ischemia and hypoxia, thereby decreasing sustained VEGF production; it offers durable efficacy but a slower onset. Seminal trials, including the Early Treatment Diabetic Retinopathy Study (ETDRS) and DRCR.net Protocol I, have established the evidence base for current management strategies: ETDRS defined diagnostic criteria and laser standards, while Protocol I validated combination therapy efficacy. Anti-VEGF combined with glucocorticoids may benefit patients who respond poorly to anti-VEGF therapy alone, but the adverse effects such as increased intraocular pressure and accelerated cataract progression limit their widespread use. The current main treatment regimen for CI-DME/PDR is centered on anti-VEGF therapy, with individualized plans optimized based on patient-specific conditions. Future research should focus on optimizing combination regimen timing, identifying suitable patient populations, the exploration of novel combination strategies (34).

The United States demonstrated a strong emphasis on anti-VEGF therapies, with a total of 69 studies (47 trial registrations and 22 publications) indexed in PubMed. In contrast, China showed a predominant focus on perioperative interventions, with a total of 40 studies (35 trial registrations and 5 publications in PubMed). These findings suggest that geographical regions may have specific priorities in therapeutic strategies, accompanied by varying rates of translation from registration to publication.

Regarding the characteristics of the study population in diabetic retinopathy research, 51.49% of studies only labeled “diabetes” without specifying the type. Since type 1 diabetes and type 2 diabetes have fundamental differences—type 1 diabetes is primarily characterized by absolute insulin deficiency, with DR progression often highly linearly correlated with disease duration; while type 2 diabetes is often associated with metabolic syndrome, insulin resistance, and chronic low-grade inflammation, and its DR may be occultly present before diagnosis—aggregating these distinct populations may introduce significant heterogeneity, weakening the external validity of the study conclusions (35). The higher proportion of studies focusing on CI-DME (29.42%) may reflect the prominence of this condition in clinical trial enrollment, as center-involving DME is the core indication for anti-VEGF therapy and the main inclusion criterion for most RCTs.

89.96% of studies set clear ophthalmic examination standards. Although most studies established inclusion criteria, a relatively small number specified clear blood glucose control requirements (179 studies, 25.32%) and other examination standards (195 studies, 27.58%). In particular, the number of studies with blood glucose control standards and other laboratory indicator control standards was insufficient; most studies defined the target population based on diabetes history and ophthalmic examination indicators. A large number of experiments focused on populations with mild to moderate vision loss, with most using >300μm as the evaluation standard for central retinal thickness assessment in patients with macular edema (77 studies, 10.89%), and some studies targeted specific ophthalmic specialty populations.

The sample sizes of diabetic retinopathy studies are generally small — 61.81% enrolled fewer than 100 participants, with studies of 1–50 participants being the most common (39.18%), while large-scale trials with over 300 participants accounted for only 13.15%. This scarcity of adequately powered studies is partly attributable to the relatively small PDR patient pool at individual centers, making it difficult for single sites to accrue more than 100 cases (36). Small samples compromise statistical power and increase the risk of type II errors, thereby undermining both internal validity and reproducibility of reported treatment effects. The establishment of multicenter collaborative networks, such as the DRCR.net series (Protocol I/T/V), RESTORE, RESOLVE, and VISTA/VIVID, is therefore essential not only to increase enrollment capacity but also to ensure sufficient power for detecting clinically meaningful effects and to enhance the generalizability of findings across diverse populations (37). Future trials should prespecify minimum sample size calculations based on clinically relevant effect sizes and incorporate interim power analyses where appropriate. Age standards are primarily focused on adults, consistent with normal research patterns. Gender standards did not impose restrictions, reflecting good awareness of external validity. Only one study was limited to males. Overall, reporting on the gender dimension is relatively standardized, but subsequent analyses should still pay attention to efficacy differences in gender subgroups. Baseline CRT values in women are usually lower than in men, which may affect the diagnostic threshold for DME and the assessment of treatment response.

Intervention timing is mostly individualized, often without fixed treatment numbers and frequencies. Notably, 37.48% of studies failed to report intervention duration, a gap that appears concentrated among records from clinical trial registration platforms (ClinicalTrials.gov and ChiCTR) rather than peer-reviewed publications, possibly reflecting insufficient rigor in registration practices. Follow-up time designs are predominantly medium to long-term, with studies of 91–365 days accounting for 47.95%. The conventional window for efficacy assessment after anti-VEGF therapy typically uses 6-month or 12-month primary endpoints, which is the main observation period design for most registered clinical trials.

Among the 707 studies analyzed herein, retinal functional indicators (represented by best-corrected visual acuity and visual field) constituted the largest proportion of primary efficacy endpoints (324 studies, 45.83%), followed by retinal anatomical parameters measured by optical coherence tomography (OCT), including central retinal thickness (CRT) and central macular thickness (CMT) (181 studies, 25.60%), with the two combined accounting for over 70%. From a pathophysiological perspective, visual acuity reflects the patient’s subjective visual benefit and is the recognized “gold standard” endpoint for anti-VEGF drug registration and approval by major international regulatory agencies (FDA, European Medicines Agency, National Medical Products Administration), while anatomical indicators such as CRT and CMT—quantified via OCT—objectively quantify the extent of retinal edema resolution, providing reproducible structural evidence for efficacy determination. Notably, functional and anatomical outcomes are only moderately correlated in DME treatment. Some patients may exhibit a “function-structure dissociation” phenomenon, characterized by a significant decrease in CRT/CMT but limited improvement in visual acuity. This discordance may reflect irreversible photoreceptor layer damage or the persistent impact of non-edematous factors (e.g., cataracts, ischemic maculopathy) on visual function.

DR progression indicators (98 studies, 13.86%) and retinal vascular/circulatory parameters (70 studies, 9.90%) constitute the second tier of the endpoint system. The former, centered on neovascular regression and DR stage improvement, reflects a fundamental modification of the disease’s natural course rather than mere symptom control, possessing irreplaceable disease-modifying evaluation value in intervention studies for PDR and severe NPDR. The latter encompasses FFA non-perfusion area and hemodynamic indicators, which can reveal improvements in the core pathological mechanism of DR—microvascular ischemia—but their application is limited by the invasiveness of FFA, the lack of standardized Optical Coherence Tomography Angiography (OCTA) quantification, and high technical thresholds. With the increasing maturity of OCTA technology and the promotion of AI-assisted quantitative analysis, vascular/circulatory parameters are expected to play a more important role as endpoints in future research (37, 38).

The distribution of secondary endpoints largely mirrors that of primary endpoints, but the reporting rates for all categories are generally higher (anatomical parameters from 25.60% to 33.95%, safety from 17.11% to 24.33%, questionnaires from 5.23% to 9.76%), indicating that researchers are attempting to incorporate more dimensional supplementary assessments beyond core efficacy evaluation, reflecting a trend towards diversification of the outcome evaluation system.

In terms of safety assessment, adverse drug reactions/adverse events are the most commonly reported component (240 studies, 33.95%), forming the core of safety evaluation. Physical examinations (intraocular pressure measurement, blood pressure, etc.) were reported in 57 studies (8.06%). For anti-VEGF intravitreal injections, although the incidence of endophthalmitis, retinal detachment, and arterial thromboembolic events is low, their consequences are severe, requiring sufficient sample sizes and long-term follow-up for reliable assessment. Furthermore, anti-VEGF drugs can still enter the systemic circulation after intravitreal injection, posing a potential threat to diabetic patients with high cardiovascular risk factors.

Among the 707 studies included in this analysis, 70.58% (499 studies) used random allocation methods, indicating that RCTs constitute the main design type for DR/DME intervention studies. Parallel group designs accounted for 75.95% (537 studies). Parallel group designs achieve baseline balance between groups through concurrent randomization, effectively controlling for time-related confounding factors (e.g., natural disease progression, seasonal fluctuations).

The implementation of blinding showed significant heterogeneity: open-label or partially blinded studies constituted the largest group (30.55%, 216 studies), whereas only 14.14% achieved double-blind design and 6.93% achieved quadruple-blind design. This pattern introduces potential performance and detection biases that threaten internal validity, particularly when outcomes include subjective measures such as visual acuity. The challenges are inherent to ophthalmic interventions — intravitreal injection involves palpable needle penetration, laser photocoagulation produces visible retinal spots, and surgical techniques are visibly distinct — making complete blinding of operators and patients technically difficult. To mitigate these concerns, future trials should employ independent masked outcome assessors (at minimum blinded central reading committees) and pre-specify objective primary endpoints (e.g., OCT-derived anatomical measures) where feasible, thereby strengthening both internal validity and result reproducibility. Single-center studies accounted for 51.34% (363 studies), revealing a decentralized, institution-based research landscape. While single-center designs offer practical advantages in implementation efficiency and investigator expertise, reliance on a single site restricts the patient pool to specific geographic, referral, and demographic characteristics, thereby constraining external validity and limiting the reproducibility of findings across different healthcare settings and ethnic populations. To strengthen generalizability, future trials should prioritize multi-center designs with deliberate geographic and ethnic diversity, and reporting should include detailed site-level characteristics (e.g., patient demographics, disease severity distribution, standard of care variations) to enable assessment of transportability. The establishment of international multicenter networks, building on models such as DRCR.net and the Asian Diabetic Retinopathy Study Group, would be particularly valuable for generating evidence applicable to diverse global populations.

Placebo controls (23.06%) were mainly seen in two scenarios: first, exploratory studies (e.g., early proof-of-concept for novel target drugs), where no established effective comparator is available; second, add-on designs for evaluating the additional efficacy of combination therapies, where an experimental intervention is added to standard treatment, and a placebo simulates the additional procedure to isolate the independent contribution of the experimental intervention. The latter is methodologically sound but requires that the background treatment is consistent across all groups, and ethical review should fully assess the risk of delayed effective treatment for patients in the placebo group.

In light of the findings presented above, we summarize the key methodological gaps identified across the included trials in **Table 5**. These gaps collectively point to systematic shortcomings in trial design, reporting completeness, and outcome selection that warrant attention in future DR interventional research.

**Table 5:** Summary of methodological gaps in included DR intervention trials.

Methodological domain	Key finding	Prevalence	Implication for trial quality
Diabetes type specification	Over half of studies failed to specify diabetes type (Type 1 vs. Type 2)	51.49% (n=364)	Potential heterogeneity in study populations; limits external validity and comparability
Glycaemic control criteria	Few studies specified clear blood glucose control requirements	25.32% (n=179)	Inconsistent baseline characterisation; may introduce confounding
Sample size	Predominance of small-sample trials; large-scale trials rare	61.81% enrolled <100 participants; only 13.15% >300	Reduced statistical power; increased risk of Type II errors; undermines reproducibility
Blinding implementation	Significant heterogeneity; open-label/partially blinded most common	30.55% open-label/partially blinded; only 14.14% double-blind	Risk of performance and detection bias; threatens internal validity
Single-center design	Majority of studies were single-center	51.34% (n=363)	Limited generalisability across healthcare settings and ethnic populations; constrains external validity
Safety outcome reporting	Adverse events most commonly reported; physical examinations less frequently documented	33.95% reported Adverse reactions/Adverse events; only 8.06% reported physical examinations	Incomplete safety profiling; may underestimate long-term adverse effects

Several limitations of this review should be acknowledged. First, PubMed was the sole bibliographic database searched; studies indexed exclusively in Embase, Web of Science, or the Cochrane Library may have been missed, although the inclusion of two major trial registries partially mitigates this concern. Second, the completeness of registry records varied considerably—for example, 37.48% of studies did not report intervention duration—which may have led to underestimation of certain methodological characteristics. Third, although data extraction and classification were performed independently by two reviewers with discrepancies adjudicated by a third senior researcher, the categorization of intervention types and outcome indicators inevitably involved a degree of subjective judgment. Fourth, consistent with scoping review methodology, no formal risk-of-bias assessment of individual studies was performed; our findings therefore describe the methodological landscape rather than the quality of the underlying evidence. Finally, because the search concluded on July 23, 2026, data for the year 2026 are incomplete, and temporal trends for the most recent years should be interpreted with caution.

## 5 Conclusion

This study systematically identifies the current landscape and shortcomings of DR intervention trials regarding study design, outcome evaluation, and safety assessment, offering guidance for the standardized conduct of future research. Across 707 studies, anti-VEGF-centered interventions, function-plus-structure endpoints, and randomized parallel-group designs predominate; yet substantial gaps persist in diabetes-type specification, glycemic eligibility criteria, intervention-duration reporting, blinding implementation, and safety documentation. Addressing these gaps through standardized registration practices, harmonized outcome sets, and complete methodological reporting will be essential to improving the rigor, reproducibility, and clinical relevance of future DR interventional research.

## Data Availability

The original contributions presented in the study are included in the article/**Supplementary Material**. Further inquiries can be directed to the corresponding author.

## Supplementary material

Supplementary Table 1 Search strategies.

Supplementary Table 2 Information of included studies.

Supplementary Table 3 Coding_Framework.
