# Supplementary material for: Mapping the methodological framework of diabetic retinopathy interventional trials: a scoping review on trial design, execution, and reporting practices
Source: Front Endocrinol (Lausanne). 2026 Oct 9;17:1961622. doi: 10.3389/fendo.2026.1961622 (PMC13690659; doi:10.3389/fendo.2026.1961622)
Supplement: Supplementary file 1 [file Table1.docx]

**Supplementary Table 1 Search strategy**

| **The search strategy for PubMed** | |
| --- | --- |
| **Number** | **Search terms** |
| #1 | ((("Diabetic Retinopathy"[Mesh]) OR (Diabetic Retinopathies)) OR (Retinopathies, Diabetic)) OR (Retinopathy, Diabetic) |
| #2 | Filters: Clinical Trial, Randomized Controlled Trial, from 2016/7/23 - 2026/7/23 |
| #3 | #1 AND #2 |

**The search strategy for** **Clinicaltrials**

(Diabetic Retinopathy OR Diabetic Retinopathies) Filters: Study Type :Interventional

**The search strategy for ChiCTR**

AREA[ConditionSearch]((Diabetic Retinopathy OR Diabetic Retinopathies)) AND AREA[StartDate] RANGE[MIN,2026-07-23] AND AREA[StudyType](INTERVENTIONAL)
